# Discovery of orally bioavailable Zika and West Nile Virus antiviral compounds targeting the NS2B-NS3 protease

**DOI:** 10.1101/2025.06.19.660642

**Published:** 2025-06-20

**Authors:** Nathaniel T. Kenton, Haim Barr, Artem Cherepakha, Lotte Coelmont, Caroline Collard, David Cousins, Geraint H. M. Davies, Oleksii Degtyarenko, Andrew Dirksen, Daniel Elbrecht, Daren Fearon, James Gayvert, Edward Griffen, Dirk Jochmans, Suzanne Kaptein, Pavlo Kliatskiy, Artem Kochetkov, Mykyta Kordubailo, Noa Lahav, Nir London, Elke Maas, Peter Marples, Nataliya Nady, Johan Neyts, Luong Nguyen, Xiaomin Ni, Yuliia Ogorodnik, Matthew C. Robinson, Carolina Turk Simpson, Ihor Tarabara, Anton Tkachenko, Annette von Delft, Frank von Delft, Oleksandr Yakymenko, Alpha A. Lee

**Affiliations:** 1. PostEra Inc, 1 Broadway, Cambridge MA 02142, USA; 2. The Wohl Drug Discovery Institute of the Nancy and Stephen Grand Israel National Center for Personalized Medicine, Weizmann Institute of Science, Rehovot 7610001, Israel; 3. Enamine Ltd., 78 Winston Churchill Street, 02094 Kyiv, Ukraine; 4. Department of Microbiology, Immunology and Transplantation, Rega Institute for Medical Research, Virology, Antiviral Drug & Vaccine Research Group, KU Leuven, Leuven, Belgium; 5. MedChemica Consultancy Ltd, Macclesfield, Cheshire, SK11 6DU, UK; 6. Diamond Light Source Ltd, Harwell Science and Innovation Campus, Didcot OX11 0QX, UK; 7. Centre for Medicines Discovery, University of Oxford, Oxford OX3 7FZ, UK; 8. Research Complex at Harwell, Harwell Science and Innovation Campus, Didcot, UK.; 9. Department of Biochemistry, University of Johannesburg, Auckland Park, Johannesburg 2006, South Africa.

## Abstract

Flaviviruses are a class of pathogenic viruses with pandemic potential which are typically transmitted via infected arthropods. In particular, Zika virus is sexually transmissible and causes congenital malformations if infection occurs during pregnancy. Although over 1.5 million people were infected during the 2015–2016 Zika outbreak, to date, there are no clinical-stage vaccines or antivirals. Herein, we report the discovery of potent inhibitors of the Zika virus NS2B-NS3 protease that also show activity against the West Nile virus NS2B-NS3 protease. Starting from a crystallographic fragment screen, we employed a pharmacophore approach coupled with high-throughput library chemistry to elaborate fragments in the active site. Potent, metabolically stable, non-covalent, non-peptidomimetic inhibitors were identified with cellular antiviral activity, with one example demonstrating excellent murine bioavailability.

## Introduction

Zika virus (ZIKV) is an important pathogen of concern for global health. ZIKV infection is usually asymptomatic in healthy adults, but in pregnant women the virus can cross the placental barrier. Fetal infection can cause microcephaly and serious neurological damage, leading to lifelong disability. During the 2015–2016 Zika epidemic in the western tropics, over 1.5 million people were infected, and an estimated 3500 children were born with disabilities.^1,2^ Although sexually transmissible,^3^ Zika is spread primarily by mosquitoes of the genus *Aedes* ^4^. Climate change increases the range of these mosquitoes,^5^ compounding the pandemic risk from increased geographical distribution, increased infection, and increased risk of relevant mutants.^5^ Other orthoflaviviruses transmitted by *Aedes*, such as dengue virus (DENV)^6^ and West Nile virus (WNV),^7^ already had a conspicuous impact even in more temperate climates. There are no ZIKV-directed antivirals approved or in clinical trials. More broadly, across the orthoflavivirus genus, only DENV-specific antivirals have been advanced to clinical trials by Janssen and Novartis, with both assets targeting the NS3–NS4B interaction.^8,9^ Therefore, the identification of broad-spectrum leads with complementary mechanisms of action is important for pandemic preparedness.

The ZIKV genome comprises a single-stranded, positive-sense RNA which encodes a polyprotein with ~3400 amino acids. During viral replication, this polyprotein is processed into three structural proteins and seven nonstructural proteins (NS1, NS2A, NS2B, NS3, NS4A, NS4B and NS5). In particular, the NS2B-NS3 protein is a serine protease, responsible for making 5 cleavages. While NS3 contains the active site and catalytic triad, it has limited activity without the NS2B protein, a membrane-bound scaffolding protein that reinforces the active conformation of NS3 via noncovalent interactions.^10^ Across orthoflaviviruses, the NS2B-NS3 protease is highly conserved, corroborating its importance in the viral life cycle. Inhibition of the NS2B-NS3 protease is expected to block viral replication, a potentially useful therapy.^11^

**Figure 1.**
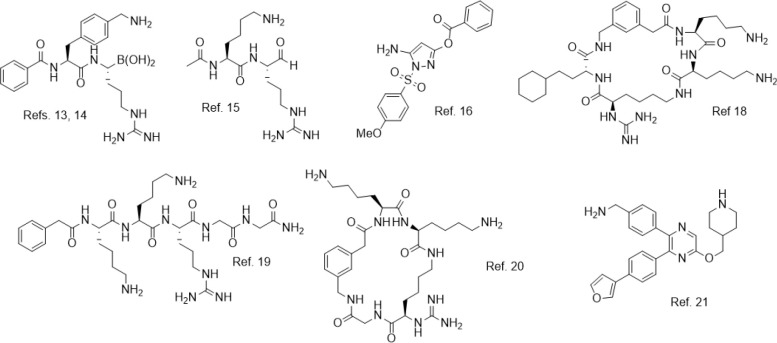
Representative ZIKV NS2B-NS3 inhibitors from the chemical literature. While many potent compounds have been reported, including some with broad-spectrum NS2B-NS3 inhibitory activity, most reported potent inhibitors are covalent, peptidomimetic, and/or polycationic.

Although protease inhibition is a precedented therapeutic mechanism for antivirals,^12^ there is thus far a dearth of advanced chemical matter directed at ZIKV NS2B-NS3. Many efforts have focused on covalent inhibitors targeting the active site Ser135 of the ZIKV NS2B-NS3 protease, including several reports of boronate esters,^13,14^ aldehydes,^15^ or electrophilic esters.^16^ However, covalent inhibitors can display promiscuous reactivity with human off-targets, thus posing a safety risk.^17^ Among non-covalent small molecules, research efforts have primarily focused on polycationic and/or peptidomimetic compounds (representative structures shown in Figure 1).^18–20^ Many described inhibitors show excellent biochemical potency, but such compounds tend to have little to no cellular antiviral activity, limiting their therapeutic utility. To our knowledge, the most advanced ZIKV NS2B-NS3 inhibitors reported to date bind an allosteric site of the protease,^21,22^ and many compounds show strong cellular antiviral activity, with one compound displaying murine *in vivo* efficacy. However, targeting allosteric sites is a known risk in antiviral drug discovery, as resistance mutations are expected to develop more quickly.^23^

Herein we report the fragment-based discovery of non-peptidomimetic, non-covalent, active site inhibitors of the ZIKV NS2B-NS3 protease, filling an important gap in the field. Starting from a crystallographic fragment screen, we used a pharmacophore approach coupled with iterative high-throughput library chemistry to elaborate fragments in the active site whilst maintaining favorable physicochemical properties.^24^ We discovered potent antivirals, with our best compound also displaying >100% murine bioavailability at a potentially therapeutic dose.

## Fragment based hit discovery with pharmacophore predictions

**Figure 2.**
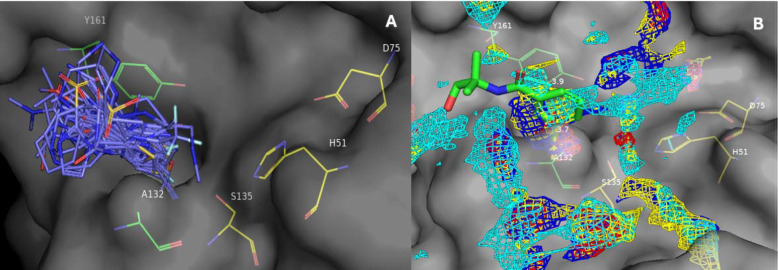
(A) Overlay of the fragments that bound the S1 site of the ZIKV NS2B-NS3 protease. The high aromatic ligandability of this site inspired compound design. (B) A proprietary machine-learning model was used to predict pharmacophore hotspots for many fragment-bound structures—the meta vector of benzamide fragment **Z68299550** (green, see also PDB ID: 7H1J) was considered an attractive growth opportunity based on this analysis (cyan mesh = aromatic, blue mesh = HB donor, red mesh = HB acceptor, yellow mesh = pos ionizable). The NS2B-NS3 catalytic triad (His51, Asp75, Ser135) is shown in yellow.

We began with a crystallographic fragment screen containing 1076 unique compounds directed at the co-expressed ZIKV NS2B-NS3 protease.^25,26^ We focused our attention on the active site of the protein, since it is hypothesized that targeting the substrate envelope in the active site of viral enzymes confers robustness to resistance.^23^ Although 46 fragments are bound near or within the active site, 34 of these were densely clustered in a deep pocket in the S1 site, sandwiched between Ala132 and Tyr161, and directly adjoining the His51/Asp75/Ser135 catalytic triad (Figure 2A). The majority (28/34) of these were aromatic compounds, supporting a notion of aromatic interactions with Tyr161. None of the fragments that were observed to bind the active site showed activity in our biochemical FRET assay up to 100 μM.

An initial pharmacophore analysis of the active site utilized machine learning to predict locations of hydrogen bond donor, hydrogen bond acceptor, positive ionizable, and aromatic pharmacophores in the active site. These predictions were overlaid on the fragment hits, allowing us to select fragments with well-positioned vectors, such as **Z68299550** (Figure 2B, PDB ID: 7H1J), which were expanded to capture untapped interactions.

**Figure 3.**
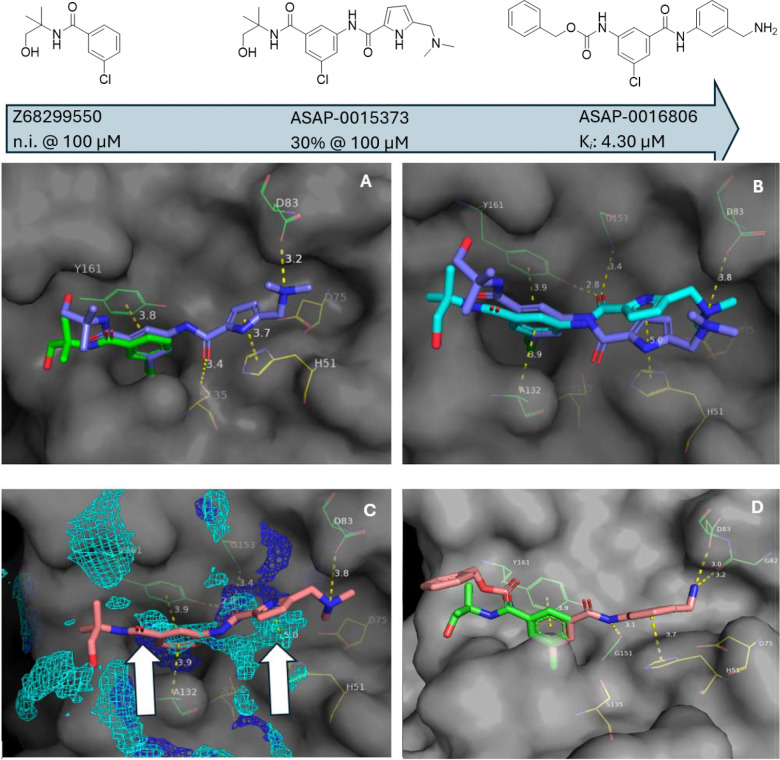
Milestones in the advancement of fragment **Z68299550** (n.i., no inhibition in biochemical assay) to inhibitor **ASAP-0016806**. (A) The docked pose of **ASAP-0015373** (purple) was predicted to span the active site and capture key interactions with His51 and NS2B residue Asp83. (B) The observed crystallographic pose of **ASAP-0015373** (cyan) showed the compound spanned the active site, but was offset from the docked pose. (C) Predicted pharmacophore opportunities in the binding site (white arrows) motivated replacement of the neopentyl amide with a more proximal hydrogen bond donor (dark blue pharmacophore mesh), and suggests an opportunity for an aromatic interaction above His51 (cyan mesh). (D) The crystal structure of **ASAP-0016806** (salmon, 7I9J) is shown overlaid with the progenitor fragment which inspired it.

Starting from **Z68299550**, we employed machine-learning-based generative techniques for library generation at the desired vector, then scored the library based on pose alignment with the predicted pharmacophore fields.^27^ These virtual libraries were triaged based on predicted binding pose with **Z68299550** as an anchor fragment. Figure 3A shows a predicted pose and potential interactions with three additional pharmacophores for one of our designs, **ASAP-0015373**, superimposed on fragment **Z68299550**. Figure 3B shows that our predicted binding mode qualitatively aligns with the solved structure. The binding mode of **ASAP-0015373**, when overlaid with the predicted pharmacophore fields around it, indicated rational avenues for optimization. In particular, aromatic and hydrogen bond donor pharmacophores were predicted, but not satisfied, by **ASAP-0015373** (Figure 3C). These predictions were leveraged to design another set of virtual libraries more closely related to **ASAP-0015373** in a second design cycle. **ASAP-0016806** emerged as a more potent inhibitor, which was crystallized with ZIKV NS2B-NS3 (Figure 3D).

Comparison of the **ASAP-0015373** and **ASAP-0016806** (7I9J) crystal structures provided a structural hypothesis for improved potency (white arrows in Figure 3C): a) replacement of the C-linked carbonyl of ‘373 with an NH-linked carbonyl of ‘806 satisfied the hydrogen-bond-donor pharmacophore that had been predicted for the former; b) reversal of the amide connecting the chlorophenyl group with the His51-targeted aromatic relaxed the linker and reduced the distance between His51 and the corresponding aromatic (5.0 Å for **ASAP-0015373** pyrrole vs. 3.7 Å for aniline of **ASAP-0016806**); at the same time, a new hydrogen bond formed between the transposed secondary amide and Gly151, and the distance between the amine substituent and the Asp83 carboxylic acid group appears to have closed (3.8 to 3.0 Å).

## Hit expansion with parallel library chemistry

**Figure 4.**
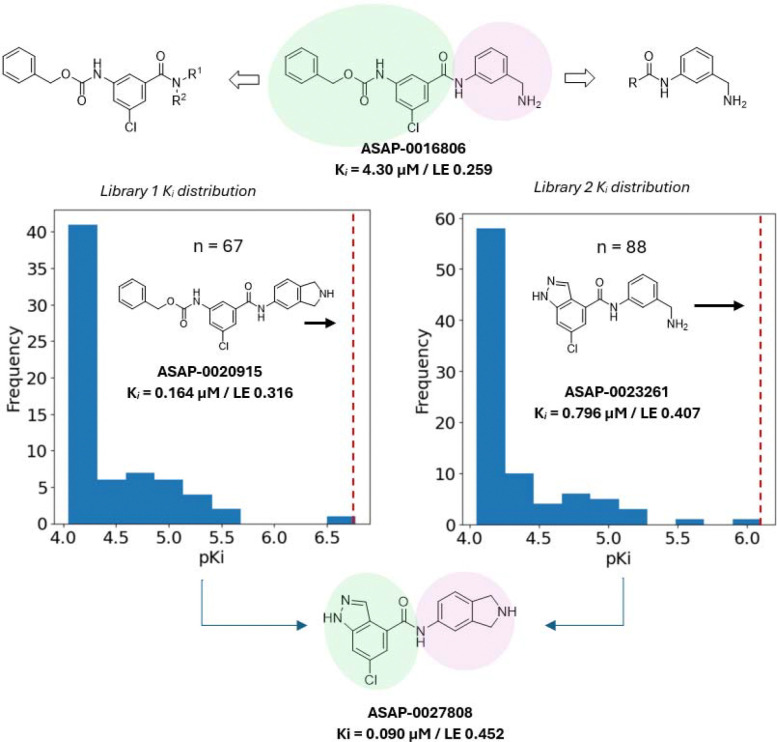
Independent libraries each maintaining one subunit of the **ASAP-0016806** structure were enumerated for hit expansion; for library 1, the green-shaded region was kept constant; for library 2, the pink-shaded region was kept constant. **ASAP-0020915** (library 1, red dotted line) and **ASAP-0023261** (library 2, red dotted line) both improved upon the ZIKV potency and ligand efficiency of the starting point. The privileged isoindoline and chloroindazole groups were then combined to generate **ASAP-0027808**, which showed a marked improvement in biochemical potency and ligand efficiency over the starting point, **ASAP-0016806**. (Ligand efficiency = p*K*_*i*_ / heavy atom count).

We next turned to expand the hit **ASAP-0016806** to increase potency. The compound’s modular structure enabled a library design based on amide coupling to vary both sides of the molecule (Figure 4). Each library identified a breakthrough compound: isoindoline **ASAP-0020915** was found in Library 1 and indazole **ASAP-0023261** was found in Library 2. Both compounds markedly improved on both the ligand efficiency (defined here as the quotient of p*K*_*i*_ and total heavy count^28^) and absolute biochemical potency of the starting point **ASAP-0016806**. The privileged isoindoline and indazole groups featured in both molecules were then combined, affording a yet more potent inhibitor, **ASAP-0027808**.

**Figure 5.**
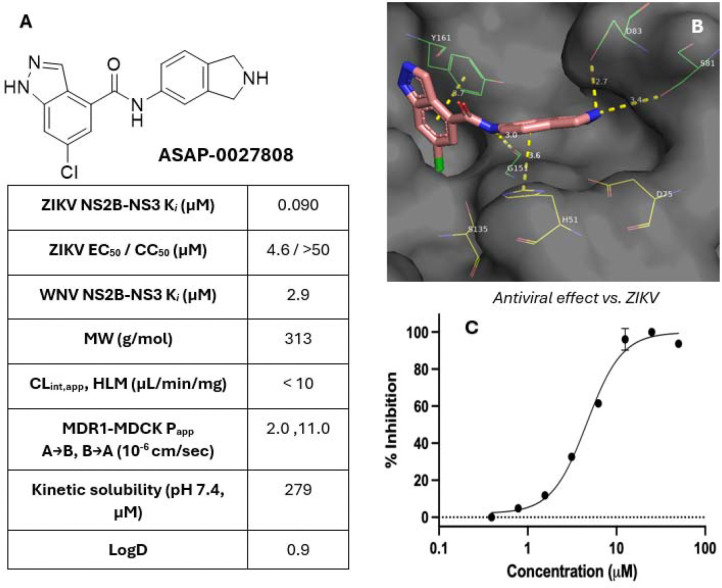
(A) Further characterization of **ASAP-0027808**. P_app_ was determined in MDR1-MDCK cells. Antiviral assays were performed in Vero E6 cells in a 7-day infection using 0.5 uM CP100356 as a P-gp inhibitor (see Supporting Information for more details). (B) The compound was successfully crystallized with ZIKV NS2B-NS3. **ASAP-0016806** and **ASAP-0027808** both appear to interact with the same residues (catalytic triad His51/Asp75/Ser135 colored yellow, other key residues Tyr161, NS2B Ser81, and NS2B Asp83 colored green). (C) Dose-dependent suppression of ZIKV-induced cytopathy in VeroE6 cells (n=2); no changes to cell viability of uninfected cells treated with the inhibitor was observed. Cells were co-treated with 0.5 μM CP100356 (as P-gp inhibitor).

We profiled **ASAP-0027808** more extensively, noting ADME properties suitable for further development, excellent selectivity over 30 human proteases in a Eurofins selectivity panel (the highest inhibitory activity observed was 21% at 10 μM against urokinase, see Supporting Information for more details), and some activity against the WNV NS2B-NS3 protease (Figure 5A), suggesting that compounds from this chemotype might be useful pan-orthoflavivirus inhibitors. Upon crystallization in the active site of ZIKV NS2B-NS3, we noted the compound participating with most of the same residues as **ASAP-0016806** (Figure 5B): an aromatic interaction with Tyr161, a hydrogen-bonding interaction with Gly151, an aromatic interaction with His51, and an electrostatic interaction with Asp83 (with a possible additional hydrogen bonding interaction to Ser81). We observed a robust and dose-dependent antiviral effect against ZIKV in Vero E6 cells treated with a P-gp inhibitor, and no apparent cytotoxicity up to 50 μM (Figure 5C).

## Core hopping led to the discovery of lead compounds

**Figure 6.**
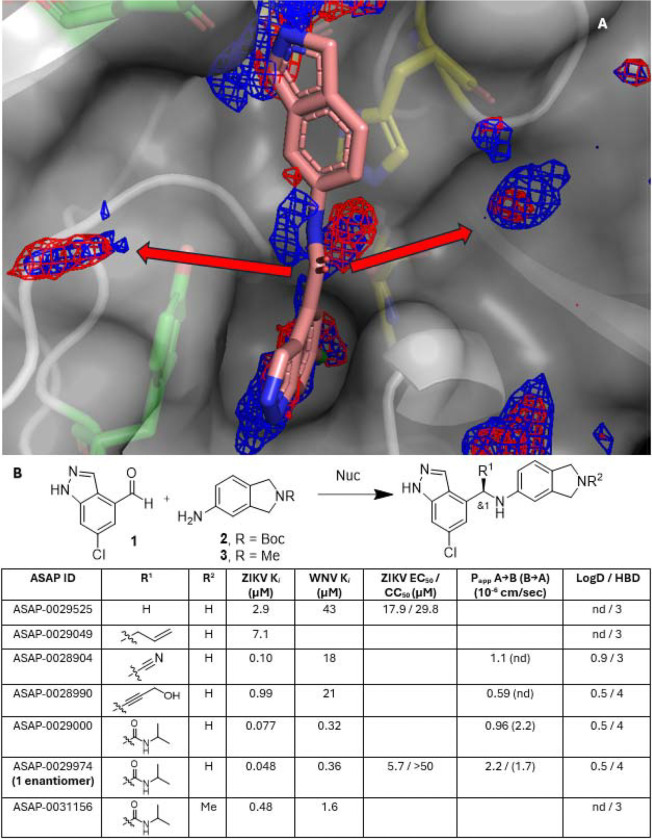
Exploring new interactions via multicomponent reactions, guided by pharmacophore predictions. (A) **ASAP-0027808** crystal structure with ZIKV NS2B-NS3 overlaid with key ML-predicted pharmacophores (red mesh = hydrogen bond acceptor; blue mesh = hydrogen bond donor). sp^2^ hybridization of the linker prevents lateral ligand growth to capture these interactions (red arrows). (B) Multicomponent reactions between compounds **1**, **2/3**, and various nucleophiles showed that sp^3^ hybridized linkers were tolerated.

Having established a connection between protease inhibition and cellular antiviral activity, we sought to further optimize potency by revisiting the predicted pharmacophores. Prediction of pharmacophores near the bound inhibitor revealed several opportunities for new polar interactions that might be captured with an sp^3^ hybridized linker rather than the amide linker (Figure 6A). Thus we hypothesized that multi-component coupling reactions between Compound **1**, Compounds **2**/**3**, and various nucleophiles might furnish a sp^3^ hybridized group which could extend laterally to exploit the predicted pharmacophores. Compounds featuring nonpolar substituents such as **ASAP-0029525** and **ASAP-0029049** were less active, in line with our pharmacophore predictions. However, polar groups were tolerated, and in particular **ASAP-0029000**, derived from isopropyl isocyanide via an Ugi reaction,^29^ showed the success of this pharmacophore-driven approach: compared to the starting point **ASAP-0027808**, it was equipotent against ZIKV NS2B-NS3, 10-fold more potent against the WNV protease, and its more potent enantiomer, **ASAP-0029474**, showed similar biochemical and antiviral potency as **ASAP-0027808** (Figure 6B).

**Figure 7.**
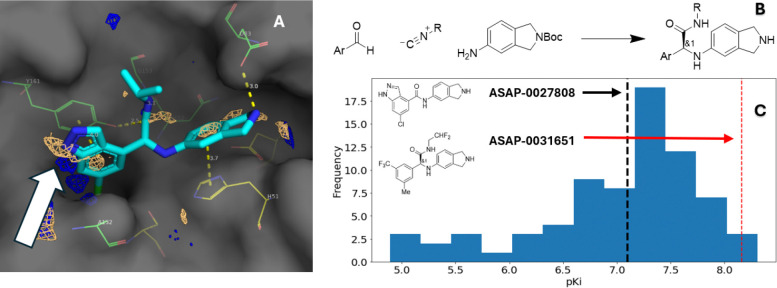
Optimization of the Ugi chemotype via library chemistry. (A) Overlapping hydrogen bond donor (blue) and hydrophobic (tan) pharmacophores were predicted at the indazole NH (white arrow), suggesting an opportunity to improve permeability by replacing a hydrogen bond donor with a lipophilic group (pdb ID: 7I9R). (B) A library that varied both isocyanide and aldehyde components was designed to explore the SAR. The isoindoline group was kept constant. (C) Results for the Ugi library; progression from lead benzamide **ASAP-0027808** (black line) to the most potent antiviral, **ASAP-0031651** (black dotted line), is indicated.

**Figure 8.**
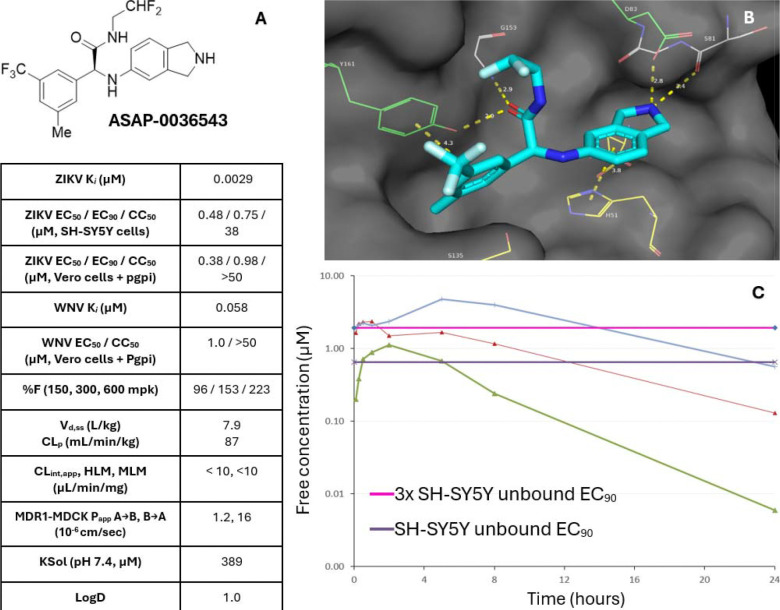
(A) Full profile of lead compound **ASAP-0036543**, the more active enantiomer of **ASAP-0031651**. (B) **ASAP-0036543** was assigned the (*S*) configuration by crystallography and the compound assumed a similar binding mode to that observed with **ASAP-0029000**. (C) Murine *in vivo* pharmacokinetics for **ASAP-0036543**. Single PO doses of 150 (green), 300 (red), and 600 mg/kg (blue) were well-tolerated and showed exposure over unbound EC_90_ (purple line) and 3x unbound EC_90_ (pink line) as determined in SH-SY5Y cells.

**ASAP-0029000** introduced a fourth hydrogen bond donor, threatening to push any follow-up compounds in the chemotype further from developable physicochemical space, especially with respect to membrane permeability.^30^ Analyzing pharmacophores predicted from the **ASAP-0029000** structure (Figure 7A), we noticed that regions defining hydrogen-bond-donor and hydrophobic pharmacophore fields overlapped near the indazole NH. This suggested an opportunity to tune the physicochemical properties of the series toward more permeable and lipophilic compounds without compromising activity. To test this hypothesis, a library based on the three-component Ugi reaction (Figure 7B) was designed to expand upon **ASAP-0029000** in which one of the primary goals was to validate this pharmacophore hypothesis. The most potent compounds from this library were also in more desirable physicochemical space, which together contributed to greater antiviral potency (see Figure 7C, 8A and Supporting Information for more details). Of the compounds in the library, **ASAP-0031651** was the most potent antiviral (*K*_*i*_ = 7.8 nM; EC_50_ = 0.45 μM, see Supporting Information).

To learn more about the new Ugi chemotype, chiral separations were performed on many of the most potent compounds from the library. In most cases, both enantiomers were active, though inhibition constants were typically separated by one order of magnitude (for one pair of examples, we observed two distinct binding modes by crystallography, see Supporting Information for more details).

**ASAP-0036543**, the most potent of the two enantiomers of **ASAP-0031651**, became our lead compound. **ASAP-0036543** displayed high biochemical potency against both ZIKV and WNV NS2B-NS3 proteases and potent antiviral activity against ZIKV in two different cell lines, i.e. VeroE6 and the neuroblastoma SH-SY5Y cells (Figure 8A). The compound had high kinetic solubility, low microsomal clearance, and acceptable permeability in an MDR1-MDCK cell line. Respectable antiviral potency against WNV in Vero cells was also noted, which had been hitherto difficult to achieve with the chemotype. **ASAP-0036543** crystallized with ZIKV NS2B-NS3 (Figure 8B) and displayed a similar binding mode to **ASAP-0029000**, with the aromatic sitting next to Tyr161, the amide carbonyl oxygen atom coordinated in a possible bidentate hydrogen-bonding pattern with Gly153 and Tyr161, and the isoindoline making key interactions with His51 (aromatic) and Asp83 (electrostatic). **ASAP-0036543** was then advanced to murine pharmacokinetic studies to assess its potential as an *in vivo* tool compound.^31^ The compound was well-tolerated at 150, 300, and 600 mg/kg oral doses (Figure 8C), with dose-dependent increases in exposure: at 600 mg/kg (blue curve), 223% oral bioavailability was observed, with free plasma concentrations remaining above 3x unbound EC_90_ for 12 h (Figure 8C, pink line) and unbound EC_90_ for 24 h (purple line). A combination of the favourable murine pharmacokinetics and potent antiviral activity of **ASAP-0036543** suggested it may be efficacious *in vivo*^32,33^.

## Conclusions

We have described a fragment-based lead discovery campaign against the ZIKV NS2B-NS3 protease, which culminated in potent cell-active compounds against both ZIKV and WNV. Our strategy combines the iterative use of machine learning predicted-pharmacophores and high-throughput chemistry. Our lead compound, **ASAP-0036543**, was tolerated in mice at doses that could cover antiviral EC90, thus it may be a suitable *in vivo* tool compound for ZIKV infection.

Further, it is noteworthy that, as the chemotype matured from the first potent hit to the lead compound, there was no commensurate increase in synthetic complexity. Both **ASAP-0016806** and **ASAP-0036543** were synthesized in two simple synthetic steps from commercially available building blocks. We hope the simple structure of the chemotype disclosed herein facilitates future efforts in lead optimization.

## Supplementary Material

Supplement 1
